# Reducing moral distress through interdisciplinary collaboration: the impact of a weekly palliative care and neonatology conference

**DOI:** 10.1186/s12904-025-01915-y

**Published:** 2025-11-11

**Authors:** Kirthi Devireddy, Riddhi Shukla, Rachel Boren, James E Slaven, Rebecca A Baker, Jayme D Allen, Karen M. Moody

**Affiliations:** 1 Department of Pediatrics, University of Texas Health Science Center- Houston, Houston, US; 2 Department of Pediatrics, Indiana University School of Medicine, Indianapolis, US; 3 Department of Pediatrics, MD Anderson Children’s Cancer Hospital, Houston, US; 4 Department of Biostatistics and Health Data Science, Indiana University School of Medicine, Indianapolis, US

**Keywords:** Moral distress, NICU, Physician, Nurse, APP, Palliative care, Non-beneficial care

## Abstract

**Background:**

Moral distress is the experience of knowing what ethically right action to take, but being unable to act accordingly, due to external factors. It is an experience common to providers working in the neonatal intensive care unit (NICU) where care for infants, often born at the edges of viability or with other life-limiting diagnoses, includes life and death medical decision-making in the context of uncertain prognoses. Palliative care, which aims to reduce suffering, can assist with staff moral distress by providing space for conversations regarding goals-of-care and end-of-life decision making.

**Methods:**

The palliative care and NICU teams co-developed a weekly, case-based conference to discuss palliative care domains of high-risk newborns including pain and symptom management, goals of care, spiritual support, and psychosocial strengths and challenges. The Moral Distress Thermometer (MDT) and the Moral Distress Scale-Revised (MDS-R) were collected at baseline and at 6- and 12-months post-intervention implementation. Quantitative and qualitative analyses were employed as appropriate.

**Results:**

One-hundred thirty-seven participants completed both surveys at baseline including 46 physician/advanced practice providers (MD/APPs) and 91 registered nurses/other health professionals (RN/OHPs). There were statistically significant improvements in both the mean MDT scores and the mean MDS-R for the overall cohort and specifically for the RN/OHP group from baseline to 12-months post-intervention. There was a trend towards improvement on these measures among the MD/APP cohort. Qualitative analysis of the free-text responses revealed several themes describing moral distress in the NICU. Themes common to both groups included: futile/non-beneficial care, prognostic uncertainty and prognostic communication, team conflict, institutional constraints and cultural bias. The theme, “End-of-life (EOL) care inconsistent with personal values” emerged among the RN/OHPs. RN/OHPs experiences were shaped by their proximity to the patient and their role as patient advocate. The MD/APP group reported more cognitive and decisional distress.

**Conclusion:**

A NICU and palliative care-weekly-collaborative conference resulted in significantly decreased moral distress among NICU staff. Qualitative data revealed that both prolonging life with life-sustaining medical therapies (LSMTs) and ending it by withdrawing LSMTs in the context of prognostic uncertainty and institutional constraints creates significant moral distress among staff. Palliative care and NICU programs should consider implementing regular interdisciplinary collaborative conferences to address this distress.

## Background

Healthcare professionals working in the neonatal intensive care unit (NICU) often face ethical challenges that can lead to moral distress. Moral distress is defined as the psychological discomfort or anguish experienced when a healthcare professional recognizes the ethically appropriate course of action but is unable to take it due to external factors such as societal, institutional, religious, or socioeconomic influences, or due to perceived inadequacies in time, training, resources, or staffing [1]. The concept of moral distress emerged from nursing literature, describing the distress nurse’s experience when their personal ethics conflicted with the medical care they were expected to provide. Over time, this concept has expanded to encompass the experiences of all healthcare team members, including physicians, when their personal ethics are challenged by patient-care decisions, influenced by institutional resources, team conflicts, and medical interventions, that may be viewed as more burdensome than beneficial [2].

The NICU environment is particularly susceptible to situations that can induce moral distress [3]. Examples include the rapidly evolving guidelines for resuscitating peri-viable neonates, difficulties in predicting survival and neurological outcomes for very low birthweight infants, and the overall high morbidity and mortality rates among medically complex and fragile newborns, which are often juxtaposed against medical interventions associated with uncertain outcomes, as well as pain and suffering [4, 5].

Although many studies indicate that healthcare professionals in the NICU experience significant moral distress, few effective interventions have been identified to mitigate it [6]. Pediatric palliative care (PPC) offers an interdisciplinary approach that addresses the physical, emotional, spiritual, and social needs of the infant and family, providing a framework for healthcare providers to develop care plans that align with parents’ values and perspectives on their infant’s best interests. PPC teams explore parents’ support systems, cultural beliefs and perceptions of illness. They serve as a sounding board for parents by creating space for them to reflect on complex medical information, articulate their hopes and values, and consider the implications of different treatment pathways. To enhance the education and perspectives of NICU faculty and staff on PPC, we developed an interdisciplinary, case-based conference focused on neonatal palliative care. We hypothesized that the conference would not only strengthen staff understanding and attitudes towards PPC but also reduce moral distress by providing a structured forum to discuss palliative care challenges from the perspectives of multidisciplinary team members. Previously, we reported positive outcomes regarding its impact on provider attitudes and knowledge [7]. In this report, we focus on the secondary outcome: moral distress.

## Methods

This study was approved by the Indiana University Institutional Review Board, which granted a waiver of consent for study participants. NICU and PPC team members organized a weekly, one-hour interdisciplinary meeting to discuss palliative care domains for high-risk newborns in the NICU, using a structured discussion tool. Eligible infants for discussion were those admitted to the Level IV academic NICU with significant morbidity and mortality based on published guidelines [7, 8], and discussion with the neonatalogy provider team and nursing leadership.Specifically, infants were eligible for discussion if they had: (1) a condition that was expected to require prolonged intensive treatment aimed at prolonging life (e.g., short gut syndrome, chronic respiratory failure, etc.), (2) a condition for which cure is unlikely (e.g. inoperable complex congenital heart disease, congenital diaphragmatic hernia with hypoplastic lungs, etc.), (3) progressive conditions without a cure (e.g. inborn errors of metabolic disease, neurodegenerative diseases expected to lead to respiratory failure, etc.), or (4) a disease causing extreme disability and health vulnerability and/or death (hypoxic-ischemic encephalopathy, severe CNS malformations, etc.). Once the list of eligible babies each week was established, each provider team, in consultation with nursing leadership, identified one for discussion that presented the greatest challenges with respect to complexity of care, aligned decision-making, and/or clarity of goals of care. Initially, four infants, one from each of our four NICU teams, were selected for discussion. However, halfway through the year, the agenda was revised to focus on two infants per meeting to allow more time for discussion. With rare exceptions, infants were discussed only one time. The entire NICU staff and all consulting faculty for each identified case to be discussed were informed in advance via secure email. Meetings were held in a designated conference room within the NICU on a consistent day and time, with the option to join remotely by phone for those unable to attend in person. Bedside staff involved in the infant’s care were personally invited to the meetings. Each session was attended by 2–5 personnel from the PPC team, including a PPC physician, (MD) advanced practice providers (APPs), a social worker, and a chaplain. On average, 12 NICU staff members participated, including neonatologists, APPs, bedside registered nurses (RNs), respiratory therapists, and support staff such as social workers, pharmacists, chaplains, family support coordinators, and child life therapists. Occasionally a physician from another subspecialty serving in a consulting role was also in attendance. A physician member of the research team was also present. The discussion of each case began with a NICU update, providing a brief overview of the medical course, including gestational age, current diagnosis and treatment, code status, anticipated need for surgical intervention, and the attending neonatologist’s prediction of survival likelihood to discharge. Following the NICU update, the study investigators led a structured discussion using a standardized template, guided by the PPC domains of pain and symptom management, goals of care, spiritual support, and psychosocial aspects, including the family’s strengths and challenges [7]. Family strengths and challenges (such as bonding, transportation, job stability, family dynamics, etc.) were explored based on care team member experience with the family. Specific palliative care principles were incorporated into the case-based discussions to enhance staff and provider knowledge of PPC. Providers reported a higher level of comfort with PPC after participation in these conferences [7]. Team members from all disciplines and medical specialties involved in each infants’ care were encouraged to attend the meeting, express concerns and provide input during the discussion.

To measure moral distress, the Moral Distress Thermometer (MDT) and the Moral Distress Scale-Revised (MDS-R) were utilized. The MDT, collected at baseline and at 6- and 12-months post-initiation of this intervention, is a self-report tool using a 0–10 scale to assess the degree of moral distress experienced in the past two weeks. Of note, the conferences continued for a total of 13 months, a full month longer than the final data collection point. The MDT has been validated and shown to be reliable among pediatric nurses [9]. A 1-point change has been shown to discriminate between nurses who have considered leaving their jobs versus nurses who have vacated their positions [9]. The Moral Distress Scale-Revised is a 21-item survey that measures the intensity (from 0 [none] to 4 [great extent]) and frequency (from 0 [never] to 4 [very frequently]) of moral distress experienced by the participant in 21 different clinical situations. An example of an item is “Feel pressure to order what I consider to be unnecessary tests and treatments.” In addition to the 21 items, space is allotted for participants to create their own items with the prompt: “If there are other situations in which you have felt moral distress, please write them and score them here.” Scores for intensity and frequency for each item are multiplied and added together resulting in a score range of 0–336. The scale also includes 2 questions regarding intentions to leave one’s current position. The MDS-R is validated and shown to be reliable among pediatric nurses, physicians, and allied health professionals working in intensive care units. MDS-R scores have been shown to be significantly higher for both physicians and nurses considering leaving their positions [10]. The clinical research coordinator (RB) de-identified all data prior to analysis. Demographics were reported with raw data and summary statistics. Baseline mean MDT and MDS-R scores for the physicians/advanced practice providers (MD/APPs) and nurses/other health professionals (RN/OHPs) were analyzed as one overall cohort, and then separately by group, looking at the change from initial assessment to the two follow-up time periods of 6- and 12-months post intervention using paired t-tests; significance was set at *p* <.05. Analyses were performed using SAS v9.4 (SAS Institute, Cary, NC). All analytic assumptions were verified. There was variation in the number of surveys answered due to staff turnover and inconsistent participation, therefore, to measure effects of our intervention, we looked only at subject-matched responses on the MDT and MDS-R before and at 6- and 12-months post intervention.

Participant-generated free text from the open-ended questions on the MDS-R were analyzed using manual coding for qualitative data [11]. The data were analyzed line-by-line to identify key concepts, which were then organized into categories through constant comparison and further explored for overarching themes [12]. Illustrative quotes from the free-text responses were selected to support the identified themes. An independent second reviewer also analyzed the qualitative data, and any differences in interpretation were resolved through consensus.

## Results

Overall, 234 healthcare staff participated in the surveys by providing demographic data and 137 participants submitted both moral distress surveys at baseline. Demographics for 137 survey respondents with completed baseline surveys and for participants that completed baseline and at least one follow-up survey (*n* = 96) are shown in Table 1. Participants represented over 10 different healthcare professions, with the largest representation from RNs. The majority of participants were female, White, in their thirties and had more than 10 years of experience. (Table 1)

**Table 1:** Participant demographics

	Participants with both surveys at baseline (*n* = 137)	Participants with both baseline and at least one follow up survey (*n* = 96)
Age	39.35 (12.46); 35 (23, 72)	40.20 (12.61); 36.5 (23, 72)
Gender		
Female	123 (90.4)	85 (89.5)
Male	13 (9.6)	10 (10.5)
Race		
American Indian	1 (0.7)	1 (1.1)
Asian	2 (1.5)	0 (0)
Black	3 (2.2)	3 (3.2)
Multi	1 (0.7)	0 (0)
Unknown	2 (1.5)	2 (2.1)
White	126 (93.3)	88 (93.6)
Hispanic	1 (0.8)	0 (0)
Profession		
APP	25 (18.3)	15 (15.6)
Chaplain	1 (0.7)	1 (1.0)
Child Life Specialist	1 (0.7)	1 (1.0)
Dietician	4 (2.2)	1 (1.0)
MD	21 (15.3)	19 (19.8)
Other	4 (2.9)	3 (3.1)
Pharmacist	1 (0.7)	1 (1.0)
RN	60 (43.8)	39 (40.6)
Respiratory Therapist	17 (12.4)	13 (13.5)
Social Worker	3 (2.2)	3 (3.1)
Years of Experience		
<1	4 (2.9)	4 (4.2)
1–3	35 (25.6)	24 (25.0)
4–6	24 (17.5)	13 (13.5)
7–10	16 (11.7)	13 (13.5)
>10	58 (42.3)	42 (43.8)
NICU deaths experienced in past year		
0–3	65 (47.5)	41 (42.7)
4–7	51 (37.2)	41 (42.7)
8–11	9 (6.6)	5 (5.2)
12–15	6 (4.4)	6 (6.3)
>15	6 (4.4)	3 (3.1)
0–3	65 (47.5)	
4–7	51 (37.2)	
8–11	9 (6.6)	
12–15	6 (4.4)	
>15	6 (4.4)	

Values are mean (standard deviation); median (range) for age and frequencies (percentages) for the categorical variables

Due to variability in the response rates across surveys and timepoints, only matched data was analyzed for each set of comparisons. This lead to different sample sizes for each comparison with considerable overlap of participants and these sample sizes are depicted in Tables 2 and 3. There were statistically significant 1-point improvements in the mean MDT scores for the overall cohort and for the RN/OHP group when baseline (T1) was compared to 12 months post intervention (T3). There was also a 1-point improvement in the mean MDT score for the MD/APP group when baseline (T1) was compared to 12 months post intervention (T3). However, this difference did not reach statistical significance (*p* =.0647) in the MD/APP group. Table 2. There was no significant difference in baseline values between the MD/APP, mean (standard deviation) = 2.72 (2.38) and the RN/OHP, mean (standard deviation) = 2.86 (2.19) groups (*p* =.7448), using all participants with non-missing baseline data.

**Table 2:** MDT mean/SD values and mean change across time by role

	Assessment interval	*N*	Mean (SD)	*P* value
ALL	T1-T2	71	T1 = 3.10 (2.31) T2 = 3.34 (2.34) Change = 0.24 (2.82)	0.4768
	T1-T3	74	T1 = 3.00 (2.37) T3 = 1.99 (2.06) Change = −1.01 (2.56)	0.0011
MD/APP	T1 - T2	24	T1 = 2.96 (2.63) T2 = 2.96 (2.39) Change = 0 (2.69)	> 0.9999
	T1- T3	30	T1 = 2.83 (2.59) T3 = 1.83 (2.42) Change = −1.00 (2.85)	0.0647
RN/OHP	T1 - T2	47	T1 = 3.17 (2.16) T2 = 3.53 (2.32) Change = 0.36 (2.91)	0.3981
	T1- T3	44	T1 = 3.11 (2.23) T3 = 2.09 (1.80) Change = −1.02 (2.38)	0.0066

T1 = baseline, T2 = 6-month study interval, and T3 = 12-month study interval

Values are means (standard deviations) for means at each time point and for change between time points, where participants had both values, with p-values from paired t-tests

**Table 3:** MDS-R mean/SD values and mean change/SD across time by role

	Assessment interval	Participants (*N*)	Mean change (SD)	*P* value
ALL	T1-T2	65	T1 = 69.05 (37.90) T2 = 59.48 (40.13) Change = −9.57 (31.64)	0.0175
	T1-T3	77	T1 = 67.30 (35.72) T3 = 54.71 (35.34) Change = −12.58 (32.71)	0.0012
MD/APP	T1 - T2	19	T1 = 59.58 (30.88) T2 = 54.16 (31.11) Change = −5.42 (27.96)	0.4091
	T1- T3	30	T1 = 59.77 (29.96) T3 = 51.67 (27.77) Change = −8.10 (23.87)	0.0733
RN/OHP	T1 - T2	46	T1 = 72.96 (40.10) T2 = 61.67 (31.11) Change = −11.28 (33.18)	0.0258
	T1- T3	47	T1 = 72.11 (38.49) T3 = 56.66 (39.59) Change = −15.45 (37.24)	0.0066

T1 = baseline, T2 = 6-month study interval, and T3 = 12-month study interval

Values are means (standard deviations) for means at each time point and for change between time points, where participants had both values, with p-values from paired t-tests

The MDS-R showed that among all participants, moral distress decreased significantly from baseline (T1) to 6 months (T2) and from baseline (T1) to 12 months (T3). This significant improvement was also noted among RN/OHPs, but the improvement in scores did not reach statistical significance among the MD/APP cohort (*p* =.0733). Table 3. There was also not a significant difference in baseline values between the MD/APP, mean (standard deviation) = 58.35 (28.51) and the RN/OHP, mean (standard deviation) = 70.99 (37.54) groups (*p* =.0551), using all participants with non-missing baseline data; although a trend towards nurses having higher moral distress at baseline was noted.

Items that scored in the top 5 for moral distress in both the MD/APP and RN/OHP groups at baseline and 12 months were nearly identical for both groups at T1 and T3 although severity and frequency of moral distress for these items were lower at T3. The MDS-R items that ranked highest for moral distress at T1 and T3 for MD/APPs and RN/OHPs are shown in Table 4.

**Table 4:** Highest ranking MDS-R item scores by group at T1 (Baseline) and T3 (12-month follow up)

MDS-*R* ITEM	MD/APP (T1)	MD/APP (T3)	RN/OHP (T1)	RN/OHP (T3)
Follow the family’s wishes to continue life support even though I believe it is not in the best interest of the child	1	1	1	1
Continue to participate in care for a hopelessly ill child who is being sustained on a ventilator, when no one will make a decision to withdraw support.	2	2	2	2
Initiate extensive life-saving actions when I think they only prolong death.	3	5	5	4
Witness other healthcare providers giving “false hope” to the family	4	4	3*	3
Watch patient care suffer because of a lack of provider continuity	6	5	3*	5
Witness diminished patient care quality due to poor team communication.	5	3	6	7^

Legend: *= tie score. ^Number 6 in this category [(RN/OHP) T3] was not otherwise ranked in the table “Feel pressure to order what I consider to be unnecessary tests and procedures.” 1 = highest moral distress score rank for that group and timepoint. T1 = baseline. T3 = 12-month follow up

There were no significant differences from baseline (T1) to 6 months (T2) or 12 months (T3) in the proportion of staff who ever “previously considered leaving” their position due to moral distress (4% v 4% v 5%; *p* =.4554). There were also no differences over time (T1 v T2 v T3) in the proportion of staff that “considered leaving it now” (16% v 19% v 10%; *p* =.1774).

### Qualitative data analysis

In response to the MDS-R question regarding “other situations in which you have felt moral distress” 14 (30%) MD/APPs and 33 (36%) RN/OHPs provided written comments. Qualitative analysis of these comments revealed five themes related to moral distress in the NICU shared among MD/OHPs and an additional theme unique to each group. While there were shared experiences expressed in the comments regarding the sources of moral distress among the MD/APP and RN/OHP groups, there were also striking differences. RN/OHPs used language and expressed stories that illustrated moral distress in more emotional terms and their experiences were shaped by their proximity to the patient and their role as patient advocate and caregiver in contrast to decision-maker. The MD/APP group reported more cognitive distress associated with prognostic uncertainty, inter-provider communication failures, and providing non-beneficial treatments in the context of terminal disease and parents as medical decision-makers. Both groups were aligned in recognizing the detriments of futile care, the institutional barriers to ethical actions, and the harm caused by fragmented, unclear and/or inequitable communication. The thematic descriptions as well as similarities and differences between the MD/APP and RN/OHP groups are shown in Table 5. A more in-depth review of the themes is described below.

**Table 5:** Qualitative analysis: themes of free text responses

Theme		Shared Themes	RN-Unique Emphasis	MD/APP-Unique Emphasis
Futile/Non Beneficial Treatment	Strong distress over continued invasive care		Close proximity to perceived suffering, secondary traumatic stress	Frustration with parent-driven decision-making resulting in delivery of futile care
Prognostic Uncertainty and Prognostic Communication gaps	Concern over lack of data, inconsistent messaging; Breakdown in team-family communication		Distress from misaligned family expectations; RNs often left to clarify, soothe, explain	Distress from having to guide families without data; MDs/APPs burdened by “ownership” of communication
Team Conflict & Breakdown of Trust	Continuity of care disrupted by turnover of service		Emotional fallout from having to carry out others’ plans	Ethical distress from inheriting others’ care plans
Misalignment of Staff and Institutional Values	Unsafe staffing, lack of support		Inexperienced staff; leaders without bedside knowledge/experience; frustration with inability to provide care at level of professional standards	Lack of autonomy in policy decisions that are perceived as negatively affecting patient care
Discrimination, Cultural Insensitivity and Judgement of Parents’ EOL Decisions	Observing cultural insensitivity and discrimination		Families judged behind closed doors	Parents mistreated, ridiculed due to their beliefs, decisions, or identity
Challenges Providing Spiritual Support	N/A		N/A	Inability to provide support for parental spiritual distress
EOL Care Inconsistent with Personal Values	Both groups experienced this to some extent		Implementing care plans which they oppose on ethical grounds	MD/APP group experienced this as team conflicts in approach to care

The first theme identified is the provision of “Futile or Non-Beneficial Treatment.” Providers describe experiencing distress when they are involved in care they think is non-beneficial and when they feel their expertise or ethical perspective is ignored by parents or other staff. In addition, RN/OHPs described the emotional toll of watching the perceived suffering of dying babies up close, whereas the MD/APP group often described their distress in terms of futility of care (e.g. providing months of life-sustaining medical therapies to babies with terminal prognoses only for them to them die afterwards). RN/OHPs also commented on the need to bring in the palliative care team when full support was provided to dying babies. Both MD/APPs and RN/OHPs relayed distress about the burden and responsibility placed on parents to make critical decisions concerning continuation of life sustaining medical therapies (LSMTs) for their children. Finally, RN/OHPs reported increased distress when parents visited infrequently and elected continued futile/non-beneficent care. Sample quotes illustrating this theme include:

- “Do everything for a baby with non-viable disease …. just to have them die a few months later” [MD/APP].
- “Continuing life support when futile because parents do not want to end the life, and medical team will not tell them that it is time. This causes high moral distress on NPs and RNs especially because they {RNs} are at the bedside doing the painful lab draws and watching the painful anasarca all day” [MD/APP].
- “Patients who have a poor prognosis, but we allow families to continue futile and sometimes painful care “[RN/OHP].

The second theme that emerged from the comments from both MD/APPs and RN/OHPs was “Prognostic Uncertainty and Prognostic Communication Gaps.” This theme highlights the MD/APP distress caused by the lack of data to support accurate prognostication in medically complex neonates and perceived challenges by all in communicating prognosis to families. The RN/OHP comments revealed distress stemming from prognostic communication gaps and the resulting confusion and emotional pain experienced by families. Furthermore, in the MD/APP comments there are multiple statements about having little to no confidence in advising families about LSMTs since the data regarding outcomes are limited. RN/OHPs, who may not always recognize the limitations in data to guide providers in shared decision-making, expressed moral distress regarding the communication of “false hope,” and failure to clearly articulate prognostic trajectories, including quality of life, to parents. Examples from the written comments include:

- “A family received a life-limiting diagnosis for their child, but I did not feel that it was presented adequately to the parents.” [RN/OHP].
- “We have many patients who are sick but we cannot provide good guidance since we do not have meaningful outcome data, short or long term. However, we expect them to withdraw life support based on this limited data.” [MD/APP].
- “Care team can’t agree on prognosis” [RN/OHP].
- “Quality of life should be discussed more often”. [RN/OHP]
- “I don’t know that withdrawal of care is presented in ways that parents understand all the time. Some providers seem to avoid that route, and not all families have the knowledge to approach it on their own”. [RN/OHP]
- “I feel many parents need more guidance that it would be the more appropriate choice to not continue care. Instead, we leave it so in their hands and it is too difficult for them to make a decision”. [RN/OHP]

A third theme that became evident among both groups was “Team Conflicts and Breakdown of Trust.” The comments included in this theme highlight how the vast differences in provider approaches to patient care and the lack of communication can lead to a breakdown in trust and consequently, moral distress. MD/APPs expressed that patient care was diminished due to poor team communication and reported feeling pressure to carry out medical plans to remove LMSTs without their direct input. These findings suggest that differences in approaches to patient care among providers contributes to moral distress which is supported by the following quotes:

- “Disagreements handling parents.” [MD/APP].
- “Poor team communication/dynamics” [MD/APP].
- “Withdraw support after change of service- decision made by previous team that now I have to carry out (without my input)” [MD/APP].

Another theme illustrated that MD/APPs experience moral distress due to “Misalignment of Staff and Institutional Values.” The RNs specifically noted staffing shortages and leadership disconnection. Institutional policies and financial constraints lead providers to feel they were unable to practice according to their moral and professional standards. There was a heavy emphasis in the RN comments on moral distress experienced from working in an environment that was perceived as inadequately staffed, staffed with inexperienced nurses and disconnected leaders, and left little time for teaching, mentoring and professional development. This sentiment is illustrated in the following comments:

- “Unsafe staffing where NPs are rounding on too many patients, or doing too many procedures, or overseeing too many residents, or not enough RT staff to provide safe and adequate care.” [MD/APP].
- “Leadership on our unit does not have current bedside experience or able to assist with patient care.” [RN/OHP].
- “Severe staffing shortages coupled with the significant shift from majority mod-expert level caregivers toward novice caregivers…[RN/OHP].
- “My moral distress is about our institution, and at times myself, not being able to give the level of care I believe we are entrusted to provide, for various reasons. [RN/OH]
- “Going home on the daily knowing I couldn’t provide the care that was required. Barriers. Staff number. Staff experience. Staff commitment.”

The fifth theme identified was “Discrimination, Cultural Insensitivity and Judgement of Parents’ EOL Decisions.” Providers remarked on their distress from observing discrimination of parents which manifested in differential treatment. They also reported distress from observing the judgement that some staff expressed towards parents’ EOL decisions when those decisions were incongruent with the personal values of the staff. This compounded the already significant emotional toll of navigating EOL care. Not only was moral distress experienced because of the conflicts in care goals that sometimes arose between providers and parents but when parents were judged by other staff for their decisions, another layer of moral distress occurred. The implications of this theme are that unfair judgments of parents, including cultural and racial/ethnic biases, compound the moral distress that occurs when caring for neonates at EOL. Comments from the MD/APPs and RN/OHPs are shown below:

- “I feel distressed when a parent is mistreated due to socioeconomic status or race/ethnicity–this can also happen in many subtle ways.” [MD/APP].
- “Families being judged for their EOL decisions, whether that is prolonging or ending life or a desire for that child to die at home” [RN/OHP].

The sixth theme that emerged in the comments specifically in the MD/APP group was “Challenges Providing Spiritual Support.” The inability to meet families’ holistic needs adds distress for providers who perceive existential suffering of families. This is illustrated with the following examples:

- “Difficulty providing spiritual support.” [MD/APP].
- “I feel distressed for not being able to provide spiritual support to the families in the face of uncertainty.” [MD/APP].

The final theme that emerged and was specific to the RN/OHP group was “End-of-Life Care Inconsistent with Personal Values” which really highlighted the moral distress RN/OHPs experience when they are implementing care plans which they oppose on ethical grounds. In contrast to the experience of moral distress resulting from over-treatment, this theme highlighted the moral distress of withdrawing LMSTs from babies which RN/OHPs thought had potential to thrive, and/or when families were perceived as not ready or not aligned with this decision. The application of withdrawal of LMSTs was viewed as sometimes arbitrary or culturally biased. Also included in this theme are comments illustrating the emotional impact of trying to provide care when orders for pain and symptoms management were insufficient. The comments included:

- “Parental desire to withdraw minimal but necessary support from baby with potential to thrive.”
- “I feel this survey and topic is more directed to the distress of those who believe we wrongly extend life when it is ‘hopeless’. There are without a doubt those times, however, I feel the opposite occurs more often - as in staff pressuring parents, and other staff, to decrease support and ‘let the patient die’. “.
- The seeming capricious application of decisions to provide care/support between pts {patients} in very similar medical circumstances … Our decisions on these kinds of patients seem arbitrary and variable. It is these situations that have caused me, and many others, significant distress”.
- “In the absence of clear objective data to help direct a course, something we often do not have for our complex neonatal patients, I think it is wrong to pressure or lead a parent to a decision they do not want…. I feel my role is to support the patient/family for the time the patient is under my care, not determine the pt’s {patient’s} course of care”.

## Discussion

This study demonstrated that weekly collaborative conferences among the palliative care and NICU team members—featuring case-based discussions on palliative care domains for critically ill neonates—were associated with reduced moral distress compared to baseline levels among NICU staff. Providing a forum to discuss the newborn’s comfort, treatment goals, family dynamics, and resources for family support offered opportunities for staff to address their ethical concerns and aspects of care that conflicted with their values. Such open conversations can serve as a mechanism for alleviating distress. Research indicates that discussing morally distressing issues can be beneficial. Open forums help individuals understand that their feelings are not unique and that such distress is unfortunately common in this line of work, potentially easing the burden on healthcare providers [13, 14].

The intentional palliative care-based discussions in the collaborative conference facilitated clarification of prognosis and goals-of-care conversations and provided in-depth discussions of the family’s psychosocial strengths and challenges. These discussions may have helped the teams to transparently develop care plans that aligned with the family’s values, highlighting the role of shared decision-making in these cases and reducing moral distress. Importantly, meaningful, comprehensive, and clarifying discussions between MD/APPs and RN/OHPs can be difficult to prioritize in the daily workflow of the busy unit. The conference helped move conversations out of silos and into an impactful collaborative space, which may have helped team members feel heard and validated. A stronger collaborative relationship between the PPC and NICU teams may have also provided an additional resource for staff for support, although this was not investigated.

It is interesting that one third of participants chose to describe “other situations where they experienced moral distress” in free-text format despite the overlap of these experiences with many of the MDS-R standardized items. This suggests that there was a need for staff to express these experiences. These free-text responses provided a deeper understanding of the moral distress experienced by the NICU staff, as well as some subtle differences between MD/APPs and RN/OHPs. Although the sources of moral distress remained stable across the study timeframe, the magnitude of moral distress related to these issues decreased. Furthermore, the additional information gleaned by the free text responses offers insight to how to further tailor moral distress interventions to different disciplines. For example, given the challenges of prognostic uncertainty and communication for MDs, standardizing implementation of neonatal palliative care communication training to fellows, faculty and APPs could be an appropriate national initiative [15]. In addition, given the specific distress related to LMST removal for some RN/OHPs, additional support for bedside staff in these situations could be provided through inclusion in shared decision-making conversations, enhanced leadership support, and incorporating more breaks for self-care in these scenarios.

The improvement in moral distress in our study was more pronounced among RN/OHPs than among MD/APPs. A similar finding was noted in a previous group that instituted a multidisciplinary ethics conference to discuss high-risk patients in the Pediatric ICU [16]. Possible explanations for the discrepant effects between these groups include the slight variations in sources of moral distress as well as perceived differences in autonomy. The parents’ responsibility in making medical decisions for their newborn, the guilt associated with “giving up,” and the availability of treatment options to prolong life, can lead parents to pursue medical interventions beyond their utility. The MD/APPs are primarily responsible for leading the process of shared medical decision-making with parents of newborns [17]. When a family’s decisions conflict with the healthcare team’s recommendations, this can lead to situations in which treatments are prescribed that do not align with the bedside nurse’s values. However, nurses must perform the daily care, communicate with family members, and implement the medical plans developed by MD/APPs, regardless of their agreement with these plans. Nurses also spend more patient-facing hours than nearly any other healthcare providers in the NICU. They play a crucial role in delivering EOL care to neonates and have direct experience with the infants’ pain and perceived suffering, which can persist for extended time periods. Nurses cited moral distress related to burdensome or aggressive care that they believed was not in the patient’s best interest, inadequate pain and symptom management, EOL care inconsistent with their values, poor communication regarding prognosis, and misaligned institutional values. These sources of moral distress have been reported for nurses working in pediatric intensive care settings as well [16].

Another significant source of moral distress affecting both groups of providers (MD/APPs and RN/OHPs) was the perception of discrimination, cultural insensitivity and judgement of parents’ EOL decisions due to socioeconomic status, race, ethnicity or differences in values. Research has shown that maternal race and ethnicity may influence perceptions of neonatal suffering, quality of life and disability [18]. Furthermore, provider’s own values, unconscious bias, and implicit assumptions may guide recommendations for LSMTs [18]. The dynamic interrelationship of these factors impacts practice patterns and subsequent outcomes that can further drive inequities and unfair narratives, or if recognized, can lead to meaningful improvements in equitable patient care. In the majority white female providers surveyed, compared to a more diverse patient population, these factors may have been an important contributor to parental perceptions of suffering and disability, treatment decisions, and subsequent provider moral distress.

The sample size for the MD/APP group was significantly smaller than the RN/OHP group, reducing power to identify statistically significant changes. Of note, the magnitude of the difference noted on the MDT (1 pt) for MD/APPs may be clinically relevant [9]. In addition, some physicians, especially when they needed to present their patients, may have felt that the interdisciplinary structure of the meeting did not offer them support but instead relied on them to provide a rationale to the staff for the medical care plan. Physicians were seen as leaders in these discussions, contributing to the discussion and receiving feedback from the interdisciplinary attendees. They provided support and validation to the staff through conversations that described their own emotional toll and internal conflicts experienced when care is largely driven by parents’ decisions The sharing of distress across disciplines was perceived by the research facilitators to diffuse tensions and unify the staff but this was not formally investigated and was case-dependent. Physicians may also have other more easily available forms of peer support built into their workflow. Many patients, especially complex neonates with relatively long length of stays, are cared for by a large percentage of the MD/APP group during their hospitalization. These colleagues have ample opportunities (e.g. during changeover, in meetings, throughout the day in shared workspaces, or during team continuity-of-care meetings) conducive to sharing concerns and discussing alternate approaches in challenging cases. Given their primary role at the bedside, often with demanding patient assignments, nursing staff may have fewer opportunities or the necessary privacy for peer-to-peer conversations throughout the day or in a moment of distress. Deliberately carving out time for these conversations seems a worthwhile endeavor; previous data has shown that regular ethics nursing huddles and debriefing with a social worker improves moral distress in critical care nurses working in adult ICUs [19]. Further research is needed to investigate the differential impact of interdisciplinary palliative care discussions on MD/APPs versus RN/OHPs to optimize support across disciplines as well as identify strategies to optimize implementation across NICU programs.

NICU MD/APPs experienced moral distress related to the provision of potentially futile medical interventions, the challenges inherent in shared decision-making (particularly when navigating the difficult balance between death and survival with significant long-term morbidity), prognostic uncertainty, team conflicts over approach, cultural biases and challenges in providing spiritual support to parents. MD/APPs have an outsized role in setting limits on intensive care and determining the futility of intensive care [17]. However, futility is not a straightforward concept, nor is it easily determined. Futility of intensive care for a medically complex newborn can be divided into quantitative futility (the probability of survival approaching zero), and qualitative futility (probability of an unacceptable quality of life). The former is laden with difficulty in precision, and the latter is exclusively values- driven [20]. Newer terms such as “non beneficial care” and “inappropriate care” have emerged in an effort to further refine the concept of futility and provide more culturally acceptable terminology, however they have not resolved the inherent challenges in its determination [21]. MD/APPs typically lead discussions with parents on the potential risks and benefits of continued intensive care, offer recommendations for treatment based on parental values and patients’ best interests and engage in ongoing shared decision-making with parents [17]. Physicians’ recognition of parents as the primary advocates for their child is grounded in both the principles of neonatal care and the dynamic conversations that occur between providers and families. However, our data illustrates they can still experience moral distress and frustration when implementing their perceptions of non-beneficial treatments.

Carter et al. identified palliative care as a key area for improvement within the NICU [22]. Despite this, fewer than half of NICUs report having access to pediatric palliative care (PPC) services [23]. Several factors contribute to the limited integration of PPC in the NICU setting. Notably, deficits in palliative care education and training among NICU providers are well-documented. Many neonatology fellows report feeling ill-prepared to deliver palliative or end-of-life care, and neonatal nurses similarly lack adequate training, though national educational initiatives have emerged to address this gap [24, 25]. Even in tertiary children’s hospitals where specialty PPC teams exist, referrals are often delayed or limited to infants who are actively dying. This hesitancy may stem from concerns that involving PPC signals a loss of hope or a shift away from curative intent. However, adopting a broader model of palliative care that is grounded in psychosocial support and symptom management, could extend PPC benefits to infants with complex or chronic conditions, uncertain prognoses, or prolonged hospitalizations, thereby providing much-needed support for families [26]. Given the ongoing gaps in PPC education, and integration, initiating a collaborative conference between a small group of PPC advocates and NICU staff could serve as a valuable starting point for expanding awareness when palliative care expertise is available. Such efforts can introduce core palliative care principles, promote alignment of treatment with goals of care, and help alleviate staff moral distress.

Limitations.

The major limitation of this study is its pre/post design without a control group, which limits our ability to draw firm conclusions. Additionally, the study was conducted at a single institution, with participant demographics that were not reflective of the broader patient population, which may affect the generalizability of the findings. In addition, participation in the study conferences was entirely voluntary and may have led to a biased sample. Another limitation is that the MDT has only been validated in pediatric and adult nurses, however, the authors of the scale have also used it in samples of multidisciplinary pediatric intensive care teams [16]. The qualitative data was based only on the subset of individuals who chose to respond to the MDS-R survey free-text item, which could have biased the data towards staff experiencing greater distress. Finally, this approach is dependent on the availability of palliative care expertise. 

## Conclusions

Moral distress in the NICU is common due to the high morbidity and mortality rates of infants and the complex ethical issues that arise when caring for fragile, high-risk and medically complex newborns. A weekly collaborative conference between palliative care and neonatal providers to discuss the care of these newborns along standard palliative care domains may help reduce the moral distress experienced by the staff. Future directions could include further tailoring of moral distress interventions to address more specifically the needs of physicians, APPs, nurses and other health professionals.

## Data Availability

The datasets used and/or analyzed during the current study are available from the corresponding author upon reasonable request and in accordance with institutional regulations.
